# Study on the Deashing of Lignite with Hydrochloric Acid/Sodium Fluoride Leaching, Assisted by Microwave and Ultrasonic Waves

**DOI:** 10.3390/ma17143537

**Published:** 2024-07-17

**Authors:** Xinming Ran, Jie Yuan

**Affiliations:** 1 College of Environmental and Chemical Engineering, Dalian University, Dalian 116622, China; ranxinming1998@163.com; 2 School of Chemistry and Materials Engineering, Liupanshui Normal University, Liupanshui 553004, China

**Keywords:** microwave-assisted, ultrasonic-assisted, coal deashing, pickling, sodium fluoride

## Abstract

This study was aimed at investigating the effects of adding sodium fluoride (NaF) and using the assistance of ultrasonic and microwave energy on the removal efficiency of ash content during the hydrochloric acid (HCl) chemical leaching process of lignite samples from Zhaotong, Yunnan, China. Chemical leaching was conducted on lignite samples from Zhaotong, Yunnan, China, under the experimental conditions of time (30–120 min), temperature (55–95 °C), microwave power (240–800 W), ultrasonic power (25–100%), and NaF addition concentration (0.2–1.2 M). The addition of NaF greatly improved the removal efficiency of ash content from lignite. Under optimized conditions, the addition of NaF increased the removal rate of ash content from lignite from 25% to 65.27%. The microwave-assisted deashing of lignite can significantly improve the deashing efficiency, with positive implications for the microstructure regulations of lignite. Ultrasonic-assisted deashing can lower the temperature for coal powder burnout and enhance the combustion performance of coal.

## 1. Introduction

As one of the major energy sources in the world today, coal makes significant contributions to industrial production and transformation [1]. Lignite is an important component of coal resources, and one of its main applications is combustion in power generation [2,3]. The mineral ash content in lignite not only reduces its calorific value during combustion but also generates a lot of dust and air pollutants. Therefore, the removal of minerals is crucial for the clean and efficient utilization of lignite. The minerals in coal exert a significant impact on its utilization efficiency, which has prompted numerous scholars to study the demineralization of coal [4,5,6]. Currently, acid treatment is the main method of removing minerals from coal. Despite having not been commercialized, this method remains a crucial technique in coal science laboratories. Research indicates that hydrochloric acid (HCl) can dissolve simple compounds like phosphates and carbonates [7], but it cannot completely dissolve clay minerals [8]. Hydrogen fluoride (HF) reacts with almost all minerals in coal except pyrite, with the majority of products being water-soluble. However, some insoluble compounds, such as calcium fluoride (CaF_2_), magnesium fluoride (MgF_2_), and sodium-containing compounds believed to be sodium tetrafluoroaluminate (NaAlF_4_), are formed when the concentration of HF exceeds that required to dissolve aluminosilicate compounds in minerals [9]. Some scholars have further studied the changes in the Fourier transform infrared (FTIR) spectra of desulfurized samples [10,11]. They have found that demineralization alters the chemical composition of the treated samples, with increased volatile matter, oxygen, and nitrogen content, significantly improved combustion characteristics compared to the original coal, and reduced sulfur dioxide emissions [12,13].

Microwaves are a type of electromagnetic wave with wavelengths and frequencies ranging from 1–300 mm and 3 × 10^2^–3 × 10^5^ MHz, respectively. The exposure of samples to microwave radiation induces molecular vibration and friction, which leads to a rise in temperature and the rapid extraction of substances. Microwave radiation can be used as an auxiliary process for chemical leaching. This was demonstrated by Jorjani et al. who obtained super clean coal using HF-nitric acid (HF-HNO_3_) as a leaching agent [14]. Different materials exhibit different characteristics when exposed to microwave radiation. Wang et al. investigated the leaching kinetics of Ni, V, Fe, and Al using organic acid (oxalic acid) and inorganic acid (sulfuric acid) as leaching solvents under microwave heating. Microwave-assisted chemical leaching can improve the extraction efficiency, shorten the extraction time, and sometimes improve the purity of extracted products [15]. Che et al. used microwave-assisted acid leaching to extract nickel from laterite ore, which achieved a recovery rate of Ni of about 90.8%, higher than the traditional water bath heating method [16]. Moreover, the selective heating ability of microwave energy can improve the leaching rate of Ni in laterite ore. Due to the presence of certain pores or cracks in the structure of coal, these channels contain water, which is a good absorber of microwave energy. The microwave irradiation treatment of coal can cause phase changes and expansion, which can generate pressure inside coal mines and may damage their structure, causing cracks [17].

In recent years, ultrasonic technology has emerged as a novel and advanced field. It harnesses the power of ultrasound to create intense cavitation and facilitate the formation and breakup of small droplets within the solution. This process effectively amplifies the surface area of the solid–liquid interface, leading to improved mass transfer efficiency. Consequently, the heightened mass transfer effect facilitates the swift entry of reactive substances from the surface of solid particles into the solution, thus expediting chemical reactions. Additionally, the mechanical and thermal effects produced by ultrasonic waves serve to further drive the reaction [18,19,20]. With the assistance of ultrasonic waves, the compounds in solid samples can dissolve more uniformly into the solvent, effectively reducing the extraction time, improving the extraction efficiency, and favoring the preservation of the activity of the extracted components. 

The hydrochloric acid–sodium fluoride (HCl-NaF) solution system can achieve the acid leaching and impurity removal effect of the mixed hydrofluoric acid–hydrochloric acid (HCl-HF) system to some extent. However, NaF has advantages over hydrofluoric acid in terms of ease of acquisition and low volatility and operational hazard. In this study, HCl was used as the leaching agent and NaF as the additive to desulfurize Zhaotong lignite through conventional heating and microwave- and ultrasonic-assisted leaching. Techniques such as industrial analysis, X-ray diffraction (XRD), X-ray fluorescence spectrometer (XRF), FTIR spectroscopy, automated surface area and pore size analysis (BET), and thermogravimetric analysis (TGA) were used. The morphology, structural changes, and combustion characteristics of lignite and its different deashing samples were systematically studied. Studying the structure and combustion characteristics of these coal samples helps enrich the knowledge of coal science and gain new insights into the structural changes and thermochemical properties of coal, further expands the range of coal utilization, and improves application efficiency. 

## 2. Materials and Methods

### 2.1. Sample Preparation

The experimental lignite was obtained from Zhaotong, Yunnan, China. The experimental chemical reagents were all of analytical grade (AR), including the concentrated HCl (Chongqing Chuandong Chemical Co., Ltd., Chongqing, China), NaF (Tianjin Aopusheng Chemical Co., Ltd., Tianjin, China), and anhydrous ethanol (Tianjin Fuyu Fine Chemical Co., Ltd., Tianjin, China). The deionized water (DI) was self-made using the Milli-Q Integral (Shanghai liding water treatment equipment Co., Ltd., Shanghai, China) water purification system. The lignite was crushed using an SS-1022 high-speed multifunctional grinder(Wuyihaina Electric Appliance Co., Ltd., Jinhua, Zhejiang, China), sieved through a 200-mesh screen, dried to a constant weight in a vacuum oven with a temperature of 105 °C, and labeled as RC for later use.

### 2.2. Single-Solution Hydrochloric Acid Leaching for Ash Removal

Under initial HCl concentrations of 1–6 M, a leaching experiment was performed with a water bath heating at 75 °C. The experiment was carried out on Zhaotong lignite with a solid/liquid ratio of 1:15. After the completion of leaching, the solution was cooled. Then, it was vacuum filtered and washed with DI until the filtrate reached pH = 7, sealed and stored after drying at 105 °C in a blast drying oven for 12 h, and named HDC for later use.

### 2.3. HCl + NaF Leaching for Ash Removal

Under an initial concentration of 5 M HCl, the range of NaF addition was 0.2–1.2 M, with an HCl/NaF ratio of 1:1. The leaching experiment on lignite was conducted at 55–95 °C for 30–120 min. After leaching, the solution was cooled and vacuum filtered. The filtrate was washed with DI until its pH was 7. The filtered solution was then dried in a blast drying oven at 105 °C for 12 h, sealed for storage, and labeled as DC for later use.

### 2.4. Ultrasonic-Assisted Leaching for Ash Removal

Coal was fully mixed with the HCl/NaF solution. The ultrasonic-assisted leaching and deashing of the solution were conducted under the probe-type ultrasonic generator YTS-1200-20 (Nanjinghanzhou Technologie Co., Ltd., Nanjing, China). The leaching duration was 30–120 min. The solution was cooled upon the completion of leaching. After that, it was vacuum filtered and rinsed with DI to achieve a filtrate pH of 7. After drying at 105 °C in a blast drying oven for 12 h, the filtered solution was sealed, stored, and named DCUS for later use.

### 2.5. Microwave-Assisted Leaching for Ash Removal

Microwave-assisted leaching and ash removal were performed using an MKG-M4HA microwave high-temperature tube furnace (Qingdao Maikewei Microwave Innovation Technology Co., Ltd., Qingdao, China). The acid-soaked mixture was microwaved at various power levels (240–800 W) and heating durations (5–20 min). After leaching, the solution was cooled, vacuum-filtered, and washed with DI to achieve a filtrate pH of 7. Subsequently, the sample was dried in a convection oven at 105 °C for 12 h, sealed for storage, and designated as DCMW for future use.

### 2.6. Analytical Methods

The ash content in coal was measured according to the slow determination method of ash in Proximate analysis of coal [21]. The procedure involved placing a crucible containing the coal sample in the constant temperature zone of a muffle furnace not exceeding 100 °C, closing the furnace door with a gap of approximately 15 mm, gradually raising the temperature to 500 °C over at least 30 min and holding it at this temperature for 30 min. In addition, the temperature was further increased to (815 ± 10) °C, and the crucible was maintained at this temperature for 1 h. 

The air-dried ash content of the coal sample was calculated according to Equation (1).

(1) $$A_{ad}=\frac{m_{1}}{m}\times 100\%$$

In the formula, *A_ad_* represents the mass fraction of the air-dried basis ash, %; *m* stands for the mass of the coal sample for general analysis experiments, g; *m*_1_ denotes the mass of residue after burning, g.

The deashing rate of the coal sample was calculated according to Equation (2).

(2) $$Deashing\;rate=1-\frac{A_{ad}}{A_{raw}}\times 100\%$$

In the formula, *A_ad_* represents the mass fraction of the air-dried basis ash, %; *A_raw_* stands for the mass fraction of the air-dried basis ash in RC, %.

The physical phases of coal before and after demineralization were analyzed using an X-ray diffractometer (XRD, Bruker D8 Advance, Ettlingen, Germany) with a scanning range from 10° to 90°, scanning at a rate of 2°/min, and a copper target. The ash after combustion was analyzed by an X-ray fluorescence spectrometer (XRF, Rigaku ZSXPrimusIII+, Tokyo, Japan). The surface morphology of the coal before and after demineralization was characterized using a scanning electron microscope (SEM, ZEISS GeminiSEM300, Jena, Germany), where an energy dispersive spectrometer (EDS) mounted was utilized for the full spectrum scanning of the sample surface. The particle size distribution of the coal samples before and after demineralization was analyzed by use of a laser particle size analyzer (Malvern Mastersizer 2000, Malvern, UK). The surface functional groups of the coal samples after demineralization were analyzed using an FTIR spectrometer (IS10, Thermo Fisher Scientific, Waltham, MA, USA) in the range of 400–4000 cm^−1^, with a resolution of 4 cm^−1^. A total of 16 scans were performed. The measurements of the specific surface area and porosity of the sample were carried out using a fully automatic BET analyzer (Micromeritics ASAP 2460, Norcross, GA, USA). Prior to testing, the samples were vacuum-degassed at 150 °C for 5 h, and the testing temperature was 77 K (under liquid nitrogen). Nitrogen adsorption–desorption testing of the samples was conducted using the data at a relative pressure (P/P0) range of 0.05–0.30, and the BET multipoint method was utilized to calculate the specific surface area (SBET) and average pore size (Vmic) of the samples. TGA (Netzsch STA449F3, Selb, Germany) was conducted by heating the samples from 30 to 800 °C at a rate of 10 °C/min in an air atmosphere to characterize the combustion performance changes in the coal samples before and after demineralization.

## 3. Results and Discussion

### 3.1. Characteristics of Coal

According to the approximate analysis results of each coal sample in Table 1, the coal was deashed by a variety of methods. The ash content of the coal samples was significantly reduced, with the highest decrease to 7.14% (originally 20.56%), and the ash removal rate reached 65.3%. The fixed carbon content increased from 33.69% to 41.85%. The reduction in ash content was mainly attributed to the effective dissolution of inorganic minerals in coal under the joint action of mixed solutions of HCl and HCl-NaF in the fields of microwave and ultrasonic energy. It is worth noting that the volatile matter content in coal increased from 41.08% to 47.6% after the ashing treatment. This is primarily ascribed to the inverse relationship typically observed between ash content and volatile matter in coal, where coal with high ash content tends to have lower volatile matter content. Consequently, removing ash content from coal will increase the relative content of volatile matter. Moreover, the removal of ash may cause corresponding changes in the structure of coal during the ashing process of coal. Such structural changes may release components originally associated with ash content to become volatile matter, which thereby increases the content of volatile matter. The analysis presented in Table 2 reveals a gradual increase in the silicon content within coal ash samples, concurrent with a general decline in the total ash mass. When considering the ash proportions indicated in Table 1, it becomes evident that the maximum extraction rates for calcium, aluminum, and iron are, respectively, 98.9%, 79.56%, and 80.4%. This suggests significant potential for the removal of these elements during the coal demineralization process.

### 3.2. Influence of Experimental Factors on Deashing Rate

#### 3.2.1. Effects of Initial HCl Concentrations on Deashing Rate of Coal Samples

**Figure 1:**
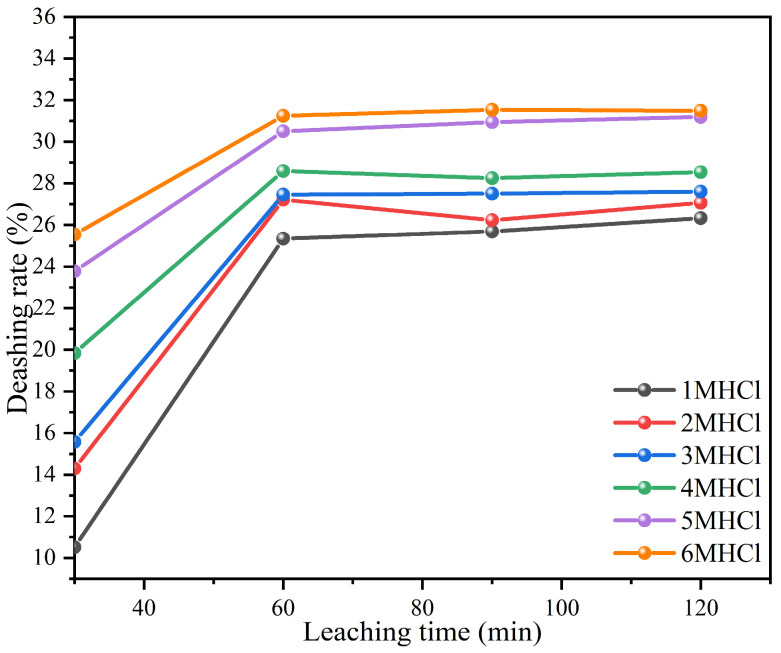
Effects of different HCI concentrations on the deashing rates of coal samples.

HCl directly affects the dissolution of acid-soluble minerals in their free form. Nevertheless, the main factor determining the solubility of these minerals is the optimal acid concentration. The effects of different HCI concentrations (1, 2, 3, 4, 5, and 6 M) on the deashing degree of coal were investigated. The results of this study are shown in Figure 1. By using only HCI leaching, a maximum deashing rate of 32.5% was achieved at an HCl concentration of 6 M. Compared to leaching with 5 M HCl, leaching with 6 M HCl gradually decreased in deashing advantage with the increase in leaching time, and the leaching rate of HCl at all concentrations approached saturation at 60 min. This may be attributable to the removal of minerals soluble in HCI, like calcite and dolomite, which left behind insoluble minerals.

#### 3.2.2. Effect of NaF Addition on the Deashing Rate of Coal Sample

**Figure 2:**
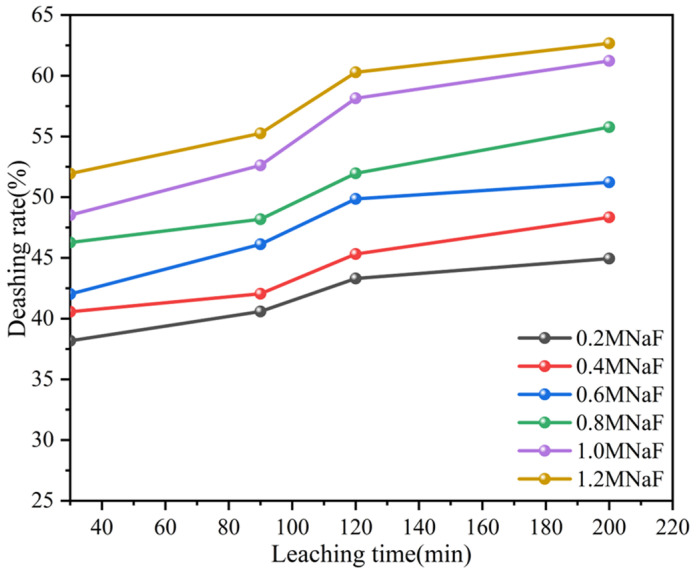
Effects of NaF addition on the deashing rates of coal samples.

During the acid leaching process of lignite, the addition of HCl as a leaching agent and NaF as an additive can markedly enhance the ash removal rate of lignite, This can be seen in Figure 2. The ash removal rate of lignite significantly increased with the addition of NaF compared to that when only HCl was added. It was speculated that the chemical reactions that may occur include:

(3) $$\begin{array}{c} {\mathrm{CaCO}}_{3}+2HCl\to {\mathrm{CaCl}}_{2}+{\mathrm{CO}}_{2}+H_{2}O \end{array}$$

(4) $$\begin{array}{c} {\mathrm{KAlSi}}_{3}O_{8}+8HCl\to 8H_{2}O+KCl+{\mathrm{AlCl}}_{3}+3{\mathrm{SiO}}_{2} \end{array}$$

(5) $${\mathrm{Al}}_{2}O_{3}\cdot 2{\mathrm{SiO}}_{2}\cdot 2H_{2}O\;+8\mathrm{HCl}\;\to 2{\mathrm{AlCl}}_{3}+6H_{2}O\;+2{\mathrm{SiO}}_{2}$$

(6) $${\mathrm{SiO}}_{2}+4\mathrm{HCl}\;\to {\;\mathrm{SiCl}}_{4}+2H_{2}O$$

(7) NaF + HCl → NaCl + HF

(8) $${\mathrm{SiO}}_{2}+4\mathrm{HF}\;\to {\mathrm{SiF}}_{4}+2H_{2}O$$

These reactions involve silicification, fluorination, chlorination, and carbonate decomposition, which contributes to the formation of the corresponding salts and water. It should be noted that these are just some possible reactions, and specific chemical reactions will be influenced by many factors, including the temperature, concentration, sample composition, etc. Based on a series of experiments and their results, the best deashing effect was achieved under the condition of 5 M HCl + 1 M NaF. Yuan et al. used HCl and NaF solutions to separate and effectively purify waste cathode carbon after alkaline melting [22]. The addition of NaF can increase the carbon content and aluminum leaching rate in the acid leaching solution to a large extent. Xue used a mixed NaF-HCl solution to treat rice straw cellulose, and the ash removal rate amounted to 96.15% [23]. The results showed that this deashing process had no significant effect on the chemical properties of cellulose. However, the crystallinity was relatively improved, as the HCl-NaF system can leach at lower acid concentrations. Huang was able to effectively extract gallium from fly ash by employing the method of NaF roasting, followed by HNO_3_ leaching [24]. 

To examine the deashing effect under optimized conditions, coal was subjected to microwave and ultrasonic leaching under the same concentration conditions.

#### 3.2.3. Effects of Different Leaching Temperatures on the Deashing Rates of Coal Samples

**Figure 3:**
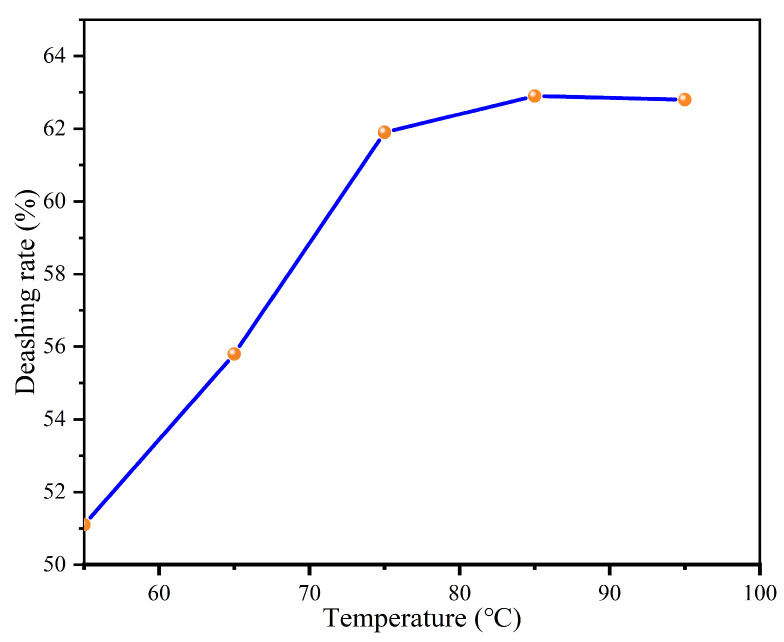
Effects of different leaching temperatures on the deashing rates of coal samples.

In the experiment, a 5 M HCl solution was used and mixed with a 1 M NaF solution for 2 h at temperatures ranging from 55 to 95 °C to investigate the effects of the solution at different temperatures on the removal of ash content from lignite. The experimental results show in Figure 3 that the ash removal rate of the lignite sample increased from 51% to 62.8% as the temperature increased from 55 to 95 °C. The increase in temperature can accelerate the acid leaching rate and improve the removal rate. This is because the increase in temperature can elevate the reaction rate and molecular motion speed. As a result, this increased the contact frequency between the reactants and the solution and the probability of effective collisions, and thus, promoted an increase in the acid leaching rate. In addition, a temperature increase can also change the equilibrium position of acid leaching reactions. Following Le Chatelier’s principle, a system in equilibrium will automatically adjust to partially counteract the effects of changes in temperature, pressure, or concentration, thus reaching a new equilibrium state. In an acid leaching reaction, increasing the temperature usually results in the absorption of heat by the system (the endothermic process) [25]. If the forward reaction (the dissolution process) is endothermic, the increase in temperature will be conducive to the forward reaction, which leads to the dissolution of more solid substances into the solution, thereby raising the solute concentration in the solution. Conversely, increasing the temperature will favor the reverse reaction if the reverse reaction (the precipitation process) is endothermic, which causes the solute in the solution to re-precipitate as a solid. As shown in Figure 3, leaching reactions are more favorable at high temperatures. The reason is that high temperatures can promote the movement of reactants toward products, thereby improving the efficiency of coal deashing.

#### 3.2.4. Effect of Liquid/Solid Ratio on the Deashing Rates of Coal Samples

**Figure 4:**
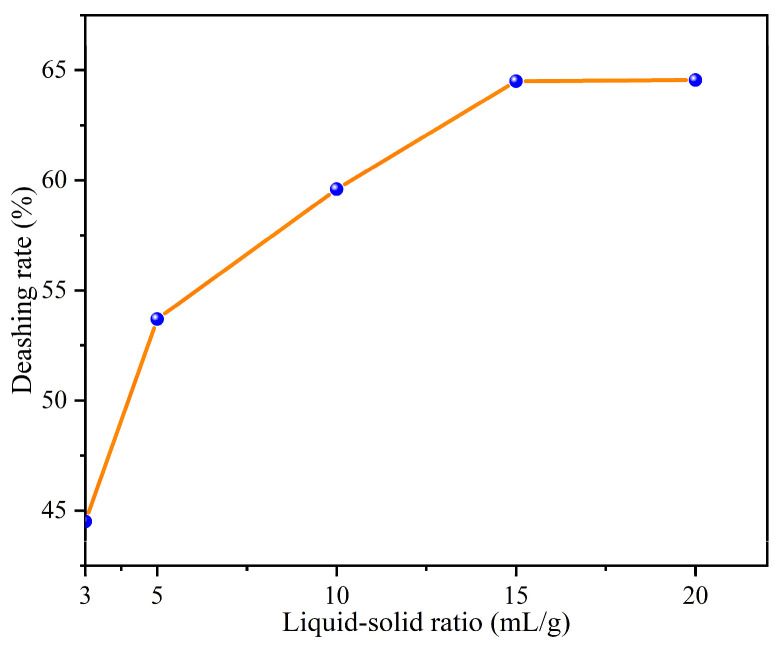
Effect of liquid/solid ratio on the deashing rates of coal samples.

In Figure 4, it is evident that the ash removal rate of coal samples increased as the liquid/solid ratio ranged from 3–15 mL/g. At lower liquid/solid ratios, the limited amount of liquid in relation to the solid results in a higher concentration of the lignite sample. This restrict the solvent’s ability to adequately reach the lignite particles, leading to an incomplete leaching process. As a consequence, some minerals may not be effectively extracted, causing a decline in the leaching rates [26]. Conversely, a higher liquid/solid ratio, where the amount of liquid is relatively greater, typically enhances the leaching rate. The increased liquid volume allows for more thorough contact between the solvent and the solid, facilitating greater solute dissolution into the liquid and resulting in a higher leaching rate. Higher liquid/solid ratios lead to a more even distribution of lignite particles in the liquid, thereby increasing the contact area and promoting the release and transfer of solute. Furthermore, the higher liquid/solid ratio improves the mixing efficiency, increasing the solute dissolution rate. The steepest slope of the ash removal rate was observed between the liquid/solid ratios of 3–5 mL/g. Beyond this range, the increase in ash removal rate became less pronounced due to mass transfer restrictions, internal diffusion limitations, and other factors [27]. Consequently, the slope of the ash removal rate with the liquid/solid ratio gradually decreased between 5 and 15 mL/g, reaching 64.5% at the liquid/solid ratio of 15 mL/g, while the increase in the liquid/solid ratio was no longer significant.

#### 3.2.5. Effect of Microwave Radiation Power on the Deashing Rates of Coal Samples

**Figure 5:**
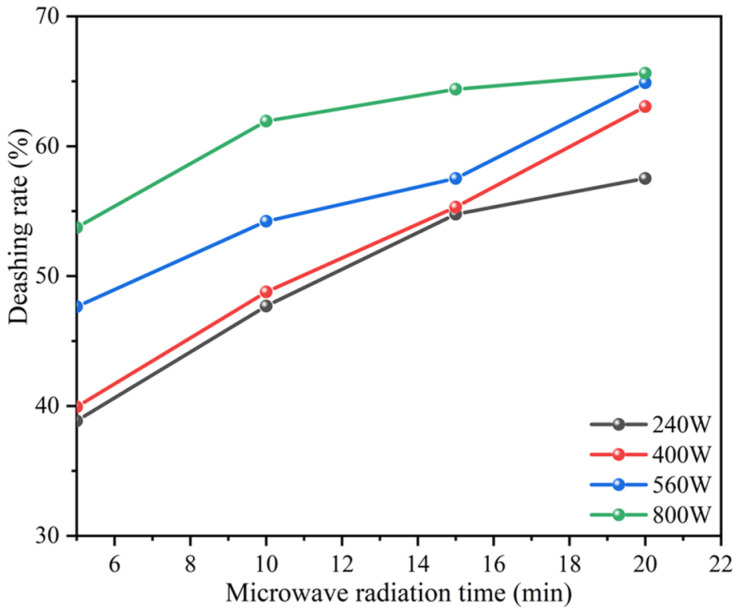
Effect of microwave radiation power on the deashing rates of coal samples.

Microwave-assisted heating significantly enhances the ash removal rate of lignite. Compared to traditional heating methods, microwave heating technology can more rapidly transfer energy directly into the material. According to the research of Özgür et al., this rapid heating helps to break the non-covalent and ionic bonds in coal, thereby increasing the selectivity of solvents for extractable substances in coal [28]. Ma pointed out, in his research, that microwaves can be absorbed by water molecules and other polar molecules in coal, producing a rapid heating effect [29]. Moisture and certain minerals in coal, such as pyrite (FeS_2_), are good absorbers of microwaves, which helps to increase coal’s ability to absorb microwaves. Microwave heating may alter the pore structure of coal, thereby increasing the effectiveness of chemical reactions. The pores and pore size of coal are crucial for its chemical deashing and ash-removal effects. Microwave heating may help to open the pores of coal, making it easier for chemical agents to penetrate the interior of coal, thereby improving the efficiency of ash removal. In Figure 5,When the amount of NaF added was 1 M, the microwave power was 800 W, and the leaching time was 20 min, the ash removal rate reached the level of leaching for two hours with 1.2 M NaF added. Therefore, the microwave-assisted chemical ash removal of coal can effectively reduce the reaction time and save energy.

#### 3.2.6. Effect of Ultrasonic-Assisted Leaching on Ash Removal Rate

**Figure 6:**
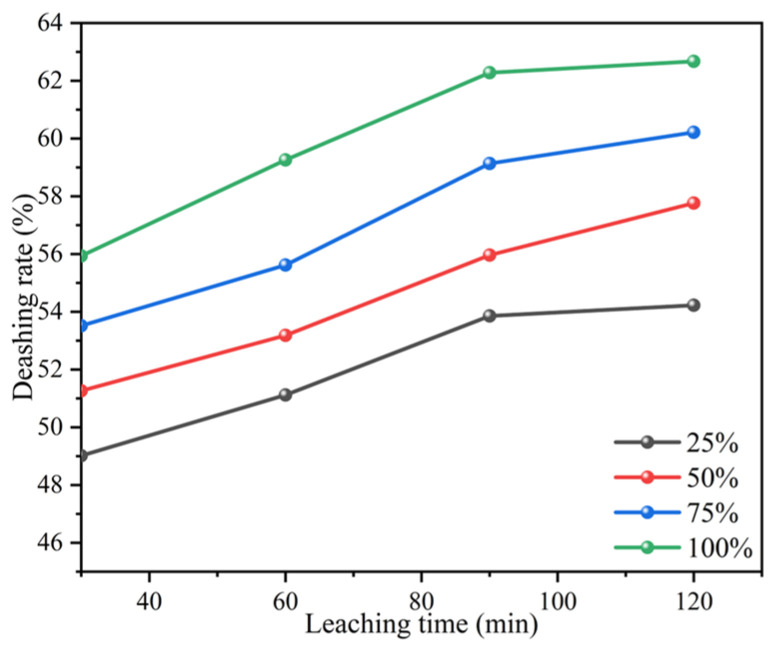
Effects of different ultrasonic power treatments of coal samples on ash removal rates.

The mechanical action of ultrasound can facilitate the mixing and contact between coal samples and solutions and increase reaction kinetics, thus ameliorating the leaching efficiency. Ultrasound enhances the rate of chemical reactions by generating cavitation effects. Research by Xiao et al. indicated that the collapse of cavitation bubbles generated by cavitation effects can produce strong impact forces and microjets in localized areas [19]. This is conducive to dispersing and cleaning the intermediate or reaction products on the surface of leaching reactants and removing passive layers on the surface. The collapse of cavitation bubbles generated by cavitation effects also increases the mass and heat transfer of the solid surface [30]. Bese explored the influence of ultrasound on copper recovery rate in the acid leaching of copper converter slag and discovered that the cavitation effects of ultrasound raised the dissolution rate of metal and the leaching rate of copper [31]. It can be seen in Figure 6 that increasing the ultrasound power can accelerate the efficiency of ash leaching.

### 3.3. Characterization of Samples

#### 3.3.1. SEM-EDS

**Figure 7:**
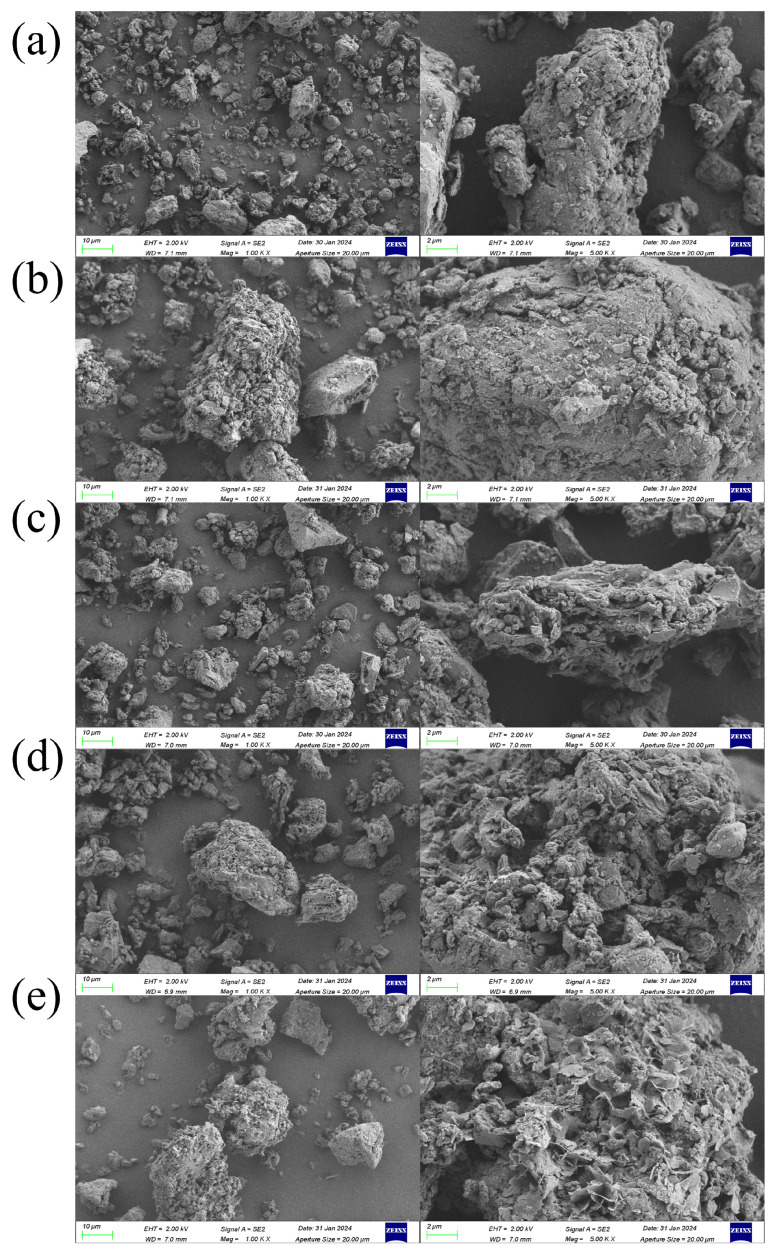
SEM and EDS images (**a**) RC, (**b**) HDC, (**c**) DC, (**d**) DCUS, and (**e**) DCMW.

Raw coal has a smooth and dense structure, with almost no pores or layered structures. Its structure is not significantly affected by HCI. As illustrated in Figure 7c, the etching effect of HCl and NaF on minerals formed a porous structure on the surface of the coal powder after the addition of NaF. This can be seen in the EDS characterization results. In Table 3, The surface of the DC showed a varying degree of reduction in elements such as Si, Al, Mg, and Fe compared to that of RC, and ultrasonic assistance helped reduce the content of Fe element on the surface of the coal powder. As demonstrated in Figure 7e, it is noteworthy that microwave assistance resulted in the formation of a greater number of layered structures on the surface of the coal powder, which may be related to the heating mode of microwave energy. Microwave heating is a non-uniform heating method of heating substances through internal friction and temperature rise [32]. During the process of microwave heating, the uneven distribution of energy gave rise to higher temperatures in some areas and lower temperatures in other areas. This non-uniform heating resulted in temperature gradients on the surface of the coal powder. Temperature gradients caused thermal expansion and contraction on the surface of the coal powder, which led to changes. Moreover, the evaporation of moisture inside the coal powder generated vapor pressure in the course of microwave heating. Vapor pressure caused bubbling and bursting on the surface of the coal powder. The rapid release of vapor led to the formation of layered structures. Furthermore, microwave heating may cause chemical changes in the organic matter inside the coal powder, which leads to the generation of gaseous and solid products. These products may form coatings on the surface of the coal powder, which results in the appearance of layered structures on the surface [29].

#### 3.3.2. Particle Size Distribution

**Figure 8:**
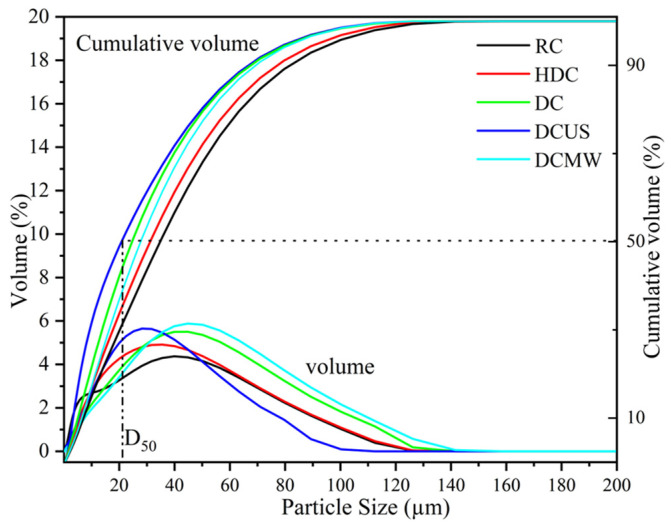
Particle size distributions of five coal samples.

The effects of different leaching methods on the particle size distribution of pulverized coal can be seen in Figure 8. D_50_ represents the particle size value at 50% of the accumulated volume, namely the median particle size [33]. In the particle size distribution curve, about 50% of the particle size is less than or equal to D_50_. HCI leaching and the addition of NaF decreased the D_50_ of particles. Notably, ultrasonic-assisted leaching had the most obvious effect on reducing the D_50_ of the coal sample particles. Xiao et al. adopted ultrasonic-assisted and traditional methods to leach spent cathode carbon (SCC) waste from aluminum electrolysis [19]. It was found that the medium-sized residue leached by ultrasonic-assisted leaching was 8.17% lower than that of the SCC sample, and 8.51% lower than that of traditional leaching residue. Ultrasonic cavitation in the liquid produced a violent cavitation effect, which resulted in the formation of small bubbles and vortices due to the rapid evaporation of the liquid. The formation and breakup of these bubbles would cause particles to break and collide, thus changing the particle size distribution. In addition, the high-energy effect of ultrasonic waves can lead to the crushing and collision of particles, which changes the size and shape of particles, thus affecting the particle size distribution. It had a positive significance in reducing the particle size of pulverized coal and improving the combustion efficiency.

#### 3.3.3. XRD

**Figure 9:**
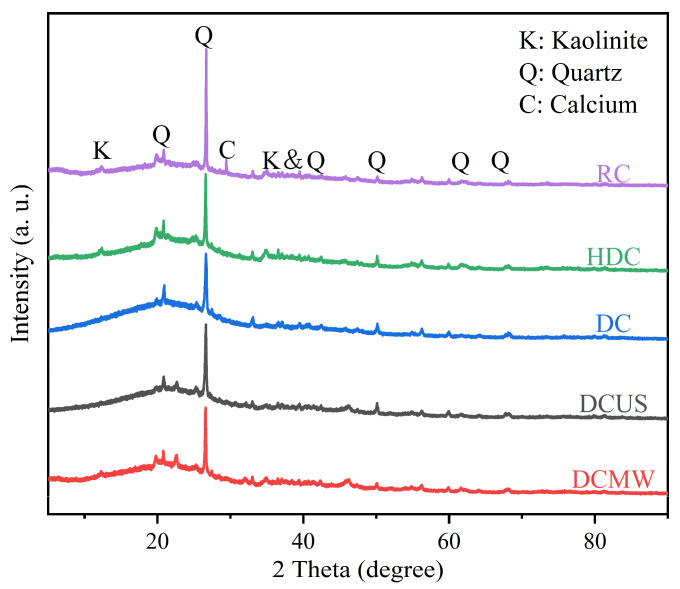
XRD patterns of five coal samples.

In Figure 9, It can be seen from the XRD diffraction peaks of the RC that quartz and kaolinite are the main mineral phases [34]. However, the maximum peak of the quartz phase was dominant in both mineral phases. The quartz phase in RC contributes most of the crystalline or free silica (SiO_2_) material, while kaolinite contributes alumina (Al_2_O_3_) and a certain amount of SiO_2_. Kaolinite [(Al_2_Si_2_O_5_(OH)_4_)], a key clay mineral in lignite, mostly coexists with illite [(K, h30) (Al, Mg, Fe)_2_ (Si, Al)_4_O_10_ [(OH)_2_, H_2_O)]], montmorillonite [(Na, Mg, Al) Si_4_O_10_(OH)_2_], and other associated minerals. These clay minerals usually exist in the coal matrix with ultrafine grains, with a particle size of less than 2 μm. In addition, peaks were also detected for calcite (CaCO_3_) and fluorapatite [Ca_5_(PO_4_)_3_F] at 2θ = 38.5° and 41.9°, respectively, as shown in the figure, which contributed substantially to a reasonable amount of oxidized minerals, like carbonates and phosphates. The diffraction patterns of the RC and HDC are similar except for the diffraction peak at 29.82°. The diffraction peak intensities of the DC, DCUS, and DCMW at SiO_2_ are significantly lower than that of the RC, which is consistent with the results shown in Table 1, which indicates that the HCl-NaF solution effectively removed a certain amount of quartz.

#### 3.3.4. FTIR

**Figure 10:**
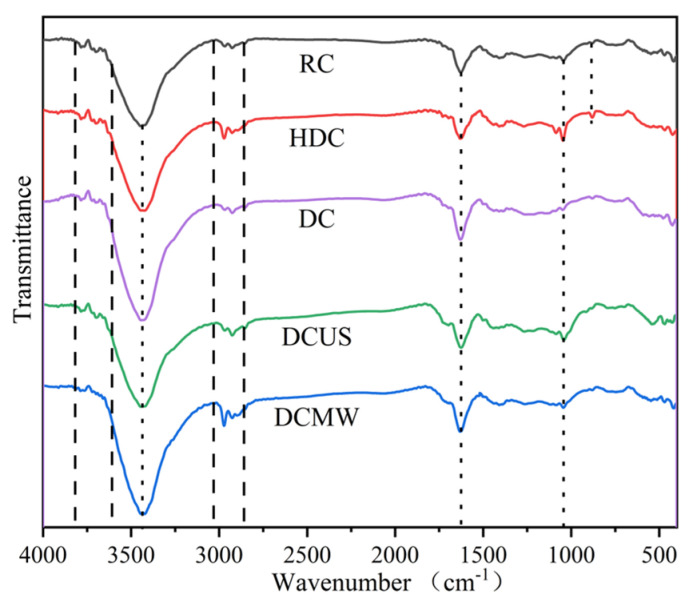
FTIR spectra of five coal samples.

In Figure 10,The weak characteristic peaks of five coal samples at 3690 and 3616 cm^−1^ are attributed to the water absorbed in clay minerals [35]. The absorption peak at 3420 cm^−1^ belongs to the stretching vibration of the hydroxyl group (-OH). The characteristic peak intensity of RC at this position is weaker than that of the other three samples. The characteristic peak of DCMW among the other three samples is the strongest, which indicates that microwave-assisted deashing can increase the content of -OH. The weak absorption band in the range of 3800~3600 cm^−1^ is interpreted as -OH bonds bound to minerals and organic matter. The absorbance band from 3000 to 2700 cm^−1^ corresponds to the stretching vibrations of aromatic and fat bonds, respectively. Because coal contains polycyclic substances, the characteristic peak of the five samples at 1626 cm^−1^ is C-C. The wide band between 1100 and 1400 cm^−1^ is due to the stretching of C-O. The characteristic peak at 1038 cm^−1^ refers to the tensile vibration of C-O-Si, and the shorter peak at 796 cm^−1^ can be attributed to the tensile vibration of Si-H. The peak intensities of the DC, DCUS, and DCWM all show decreases of different degrees, which indicates that HCl-NaF destroys the structures of C-O-Si and Si-H, while the characteristic peaks of HDC in these two places are enhanced because HCl releases silicon oxides by destroying the structures of kaolinite and other minerals.

#### 3.3.5. BET

**Figure 11:**
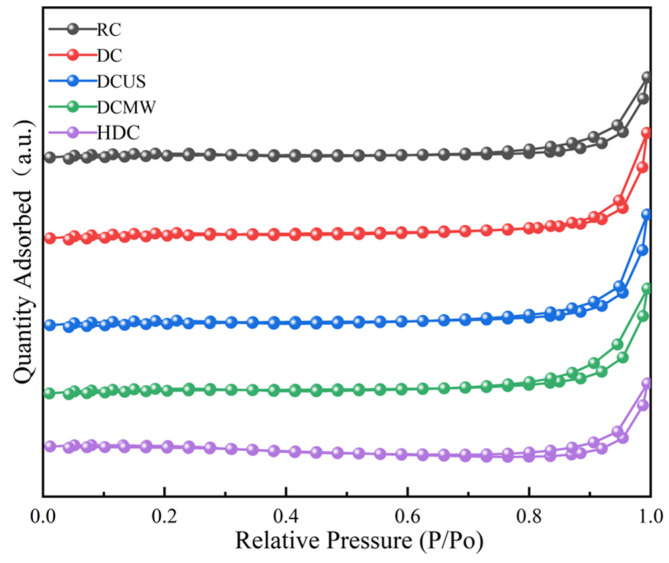
N_2_ adsorption and desorption curves (77 K) of five coal samples.

**Figure 12:**
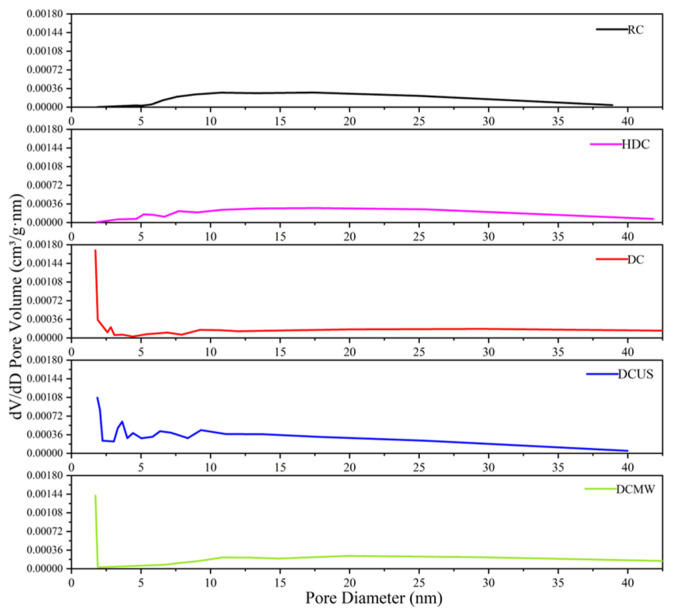
Aperture distribution of five coal samples.

Figure 11 displays the N_2_ adsorption isotherms of five coal samples at 77 K. The BET-specific surface areas and pore size distributions of the five samples were calculated based on the adsorption isotherms of N_2_ at 77 K. From the N_2_ desorption curves and pore size distributions of the five coal samples, it can be seen that deashing treatment effectively affected the specific surface area and pore size distribution of the pulverized coal [36]. The BET surface area, pore volume, and average pore size of each sample are shown in Table 4. The surface area BET of the RC was smaller than that of the other four deashed samples, which indicates that the deashing increased the specific surface area by dissolving minerals in the coal structure. The pore size distribution curve is shown in Figure 12, which clearly shows the evolution of the pore structure after different methods of deashing. After the HCl treatment, the pore size distributions of the coal samples showed no significant changes, which is associated with the limited types and quantities of minerals that HCl can dissolve. Meanwhile, the table shows that the BET specific surface area of the HDC was the smallest, which may have been due to the incomplete dissolution of minerals by HCl. The specific surface area of DCUS in the sample was slightly smaller and its pore size distribution tended to concentrate on large pores, which is probably due to the collapse of the surface structure of pulverized coal caused by ultrasonic energy.

#### 3.3.6. TGA

By analyzing the data of the TG-DTG curves, specific combustion parameters were obtained, and the influence of different deashing processes on the combustion reactivity of coal was determined. Several basic combustion parameters can be obtained from the TG-DTG curves: the ignition temperature Ti (°C), peak temperature Tp (°C), and burnout temperature Tf (°C) [37,38]. Ti represents the temperature where the weight loss rate of the coal is 1%/min, which indicates that the coal sample started to burn, which was determined by the TG-DTG tangent method. Tf stands for the temperature at which the mass loss of the coal is over 99%, which suggests that the coal ceased combustion. Tp denotes the temperature corresponding to the peak of DTG spectra, and the weight loss rate of coal reached its maximum value at this temperature.

**Figure 13:**
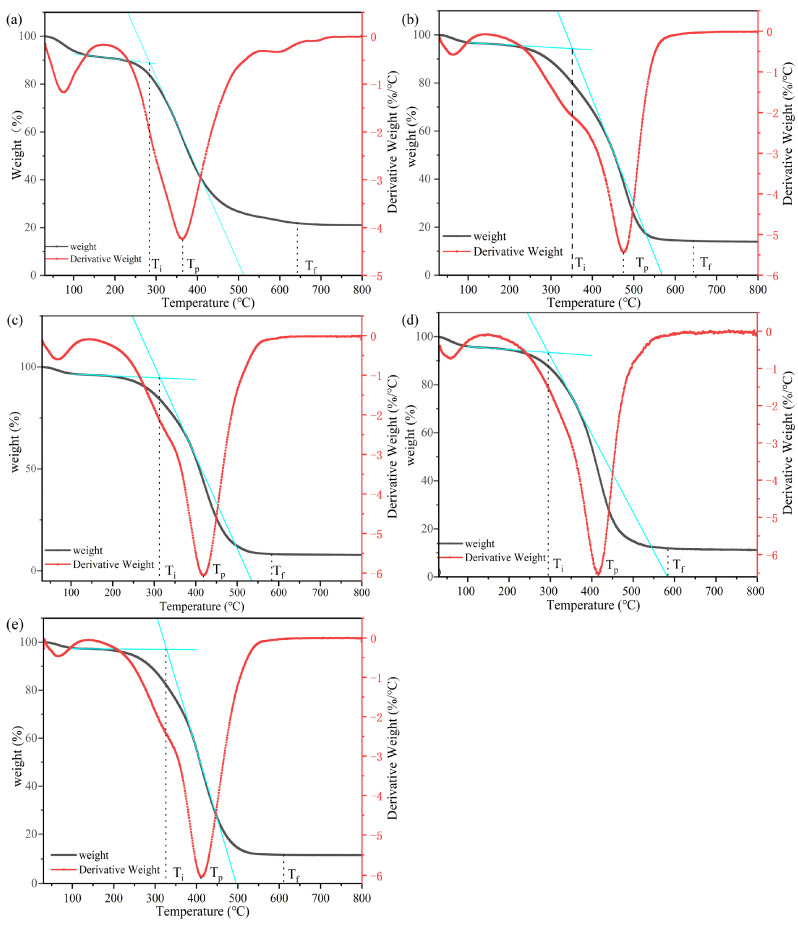
Thermogravimetric-Derivative thermogravimetric (TG-DTG) curves, (**a**) RC, (**b**) HDC, (**c**) DC, (**d**) DCUS, and (**e**) DCMW.

The combustion performance of coal is influenced by the coal quality, volatile matter content, fixed carbon content, mineral content, particle size, and oxygen concentration. Based on the same burnout procedure for raw coal and coal with multiple chemical deashing processes, the effects of different chemical deashing processes on the combustion performance of coal samples were observed. These can be seen in Figure 13. All coal samples had a mass loss of 3–7% at around 100 °C, which was due to the loss of water from the coal. When the temperature rose above 400 °C, the quality of the DC, DCUS, and DCMW exhibited sharp drops owing to the rapid combustion of the coal. The Ti values of the clean coal samples obtained by different chemical deashing processes were different. The ignition temperature of the RC was the lowest. During the combustion process, the volatile matter was first released from the coal sample for pyrolysis, followed by the burning of the coal. Because the volatile matter content in the RC was lowest, it was the first to burn, which is consistent with the approximate analysis results. In addition, the Ti of the DCUS was lowest among the four deashed coal samples, which is probably related to the effective reduction of the particle size of the DCUS assisted by ultrasound [39]. The finer particle size increased the area of contact with air and accelerated the ignition process. Hence, it can be seen that ash removal increased the ignition temperature and the thermal stability of the coal sample. 

In Table 5,With the removal of the mineral ash, the highest combustion rate temperature (Tp) of the coal sample increased, which indicates that the combustion reactivity of the coal sample decreased slowly, and the combustion reactivity of the DCMW was lowest. This is because most minerals in coal can promote the combustion of coal [40]. Thus, the combustion reactivity of coal samples became weaker, and the maximum combustion rate decreased with the removal of these minerals by acid leaching. This indicates that the correlation between the peak temperature and the mineral content of the coal was weak. The peak temperature of coal is closely linked to the content of volatile matter, which is negatively related to the peak temperature. Kizgut et al. discussed the effect of chemical deashing on the thermal behavior of bituminous coal and found that chemically deashed coal was significantly more reactive than raw coal [41]. 

Zou et al. discussed the effect of catalysts on the combustion reactivity of anthracite and reported that the presence of minerals inhibited the burnout of anthracite [42]. It can be seen in the table that the lower the ash content in the coal sample was, the lower the Tf value was. This is in line with a previous study [43]. The reason is that the minerals, clay minerals, and quartz in the coal structure had a negative impact on coal combustion and suppressed the carbon–oxygen reaction, which increased the burnout temperature of the coal samples. 

Therefore, a deashing treatment can effectively improve the thermal stability of coal, reduce its burnout temperature, and raise its combustion performance.

## 4. Conclusions

In this study, microwave and ultrasonic energy were used to assist with the HCl-NaF leaching of Zhaotong lignite. The following conclusions were drawn. 

(1) The ash content of Zhaotong lignite can be reduced to 7.14%, and the ash removal rate of Zhaotong lignite can be increased from 23% to 65.27% by optimizing the conditions of the mixed HCl-NaF solution system. The addition of NaF can effectively remove part of the silicon oxide. 

(2) Based on shortening the reaction time and saving energy, microwave-assisted deashing can change the structure of pulverized coal. Thus, it not only has a huge gain effect on auxiliary ash removal but also advantages that conventional heating does not have in controlling the structure of coal-based carbon materials. 

(3) The combustion performance of Zhaotong lignite was significantly improved by ash removal, and ultrasonic-assisted ash removal improved the combustion performance of lignite by reducing the particle size of pulverized coal.

## Figures and Tables

**Table 1:** Proximate analysis results of coal samples.

Sample	M (mass%)	A (mass%)	V (mass%)	FC (mass%)
RC	4.73	20.56	41.02	33.69
HDC	4.96	15.51	42.54	37.00
DC	5.08	7.29	46.59	41.04
DCUS	4.63	7.59	45.03	42.75
DCMW	3.41	7.14	47.60	41.85

RC, raw coal; HDC, hydrochloric acid-leached coal sample; DC, coal sample leached by hydrochloric acid and hydrofluoric acid; DCUS, ultrasonic assisted leaching of coal samples; DCMW, microwave-assisted leaching of coal samples. M, water; V, volatile matter; A, ash; FC, fixed carbon.

**Table 2:** XRF analysis of coal ash samples (mass%).

Sample	O	Mg	Si	S	Ca	Al	Fe	Ti
RC	45.7402	2.4151	16.908	3.4679	9.0946	12.7097	6.0602	1.2319
HDC	47.7417	1.0195	26.2629	1.0518	1.4451	16.1741	4.3891	1.8473
DC	47.7118	0.5559	28.0138	0.0575	0.3234	7.547	3.5246	1.9284
DCUS	48.2521	0.5245	28.21	0.0281	0.2578	7.3517	3.4142	1.9198
DCMW	49.2173	0.5631	29.5211	0.0609	0.3696	7.3517	3.3947	2.8798

O, oxygen; Mg, magnesium; Si, silicon; S, sulfur; Ca, calcium; Al, aluminum; Fe, iron; Ti, titanium.

**Table 3:** Contents of surface elements of five coal samples (wt %).

	Carbon (C)	Oxygen (O)	Silicon (Si)	Aluminum (Al)	Magnesium (Mg)	Sulfur (S)	Calcium (Ca)	Ferrum (Fe)	Fluorine (F)	Natrium (Na)	Total
RC	79.92	16.93	0.4	0.49	0.19	1.07	0.64	0.35	-	0.01	100
HDC	77.49	20.02	1.08	0.85	0.07	0.47	0.01	0.32	-	-	100
DC	80.26	18.49	0.15	0.14	0.02	0.52	0.11	0.02	-	0.03	100
DCMW	74.57	22.47	0.44	0.65	0.05	0.64	0.57	0.09	-	0.52	100
DCUS	79.13	15.46	0.98	0.72	0.31	1.12	0.67	-	1.67	0.03	100

**Table 4:** Surface area BET, pore volume, and average pore diameters of five coal samples.

	SBET (m^2^/g)	Pore Volume (cm^3^/g)	D (nm)
RC	2.4	0.017184	28.4889
HDC	4.2	0.021689	20.5253
DC	5.5	0.028410	20.8188
DCUS	4.9	0.028844	23.3291
DCMW	6.0	0.025766	17.1312

**Table 5:** Ti, Tp, and Tf of five coal samples.

	T_i_ (°C)	T_p_ (°C)	T_f_ (°C)
RC	288	363	643
HDC	351	476	600
DC	312	417	580
DCUS	293	415	582
DCMW	324	412	559

## Data Availability

The original contributions presented in the study are included in the article, further inquiries can be directed to the corresponding author.
